# Account of a Very Remarkable Chalybeate Spring, Lately Discovered near Reading

**Published:** 1804-09-01

**Authors:** J. Douglas


### Account of a very remarkable Chalybeate Spring, *lately discovered near Reading. Communicated by* Dr. J. Douglas.

MINERAL Springs of a chalybeate nature are by no means uncommon in this island; but very few, either in strength or efficacy, approach the Caversham Spa. It is situated upon a hill, about a mile from the Thames, on the Oxfordshire side of the river, and nearly two miles from Reading; a number of springs arise in the neighbourhood, but none perceptibly possessing any chalybeate property. When first discovered, it was a muddy pool covered with an ochreous crust reflecting a variety of colours. The proprietor erected a pump to exclude the atmospheric air; since which time, the water, when first drawn, has been clear and sparkling.

The smell immediately detects sulphureous gas, but it is not so strong as to give a decided character to the water. To the taste there is a very strong astringency, which remains for a considerable time upon the palate. It instantaneously produces a very deep black with infusion of galls or tea. Exposed for a short time it loses its brilliancy, and in a few hours becomes muddy, most probably from the escape of carbonic acid gas by which the iron is supposed to be held in solution. In less than two days all the iron is deposited, as the tests no longer detect it in the water. From a gallon, thirty-two grains of solid contents were procured, the greater part of which seemed to be an oxyd of iron.

It would be unnecessary and presuming, to point out to medical men the particular complaints and constitutions in which a mineral water of this nature proves useful. In many diseases of debility the most striking effects have been evinced. Though little more than a year since its first discovery, its fame has extended to the whole surrounding country, and the credulous may be daily informed of the wonders atchieved by this powerful medicine. It is not necessary that we should believe exaggerated report, as authentic evidence exists in a great variety of cases where it has been eminently useful.

Much caution is necessary in its exhibition. It is highly stimulant, as a quarter of a pint will in many induce feverishness and head-ach, and that almost instantaneously; the dose should therefore be at first small, and afterwards regulated according to its effects. It often confines the bowels, and frequently operates by urine. To be drank with greatest advantage, it should be had fresh from the pump, and then there cannot exist a doubt of its being superior in strength to the celebrated Tunbridge chalybeate.

*August* 12, 1804.

